# Observation of B$^0$ $\rightarrow$ $\uppsi$(2S)K$^0_\mathrm {S}\uppi ^+\uppi ^-$ and B$^0_\mathrm {s}$ $\rightarrow$ $\uppsi$(2S)K$^0_\mathrm {S}$ decays

**DOI:** 10.1140/epjc/s10052-022-10315-y

**Published:** 2022-05-31

**Authors:** A. Tumasyan, W. Adam, J. W. Andrejkovic, T. Bergauer, S. Chatterjee, K. Damanakis, M. Dragicevic, A. Escalante Del Valle, R. Frühwirth, M. Jeitler, N. Krammer, L. Lechner, D. Liko, I. Mikulec, P. Paulitsch, F. M. Pitters, J. Schieck, R. Schöfbeck, D. Schwarz, S. Templ, W. Waltenberger, C. -E. Wulz, V. Chekhovsky, A. Litomin, V. Makarenko, M. R. Darwish, E. A. De Wolf, T. Janssen, T. Kello, A. Lelek, H. Rejeb Sfar, P. Van Mechelen, S. Van Putte, N. Van Remortel, E. S. Bols, J. D’Hondt, A. De Moor, M. Delcourt, H. El Faham, S. Lowette, S. Moortgat, A. Morton, D. Müller, A. R. Sahasransu, S. Tavernier, W. Van Doninck, D. Vannerom, D. Beghin, B. Bilin, B. Clerbaux, G. De Lentdecker, L. Favart, A. K. Kalsi, K. Lee, M. Mahdavikhorrami, I. Makarenko, L. Moureaux, S. Paredes, L. Pétré, A. Popov, N. Postiau, E. Starling, L. Thomas, M. Vanden Bemden, C. Vander Velde, P. Vanlaer, T. Cornelis, D. Dobur, J. Knolle, L. Lambrecht, G. Mestdach, M. Niedziela, C. Rendón, C. Roskas, A. Samalan, K. Skovpen, M. Tytgat, B. Vermassen, L. Wezenbeek, A. Benecke, A. Bethani, G. Bruno, F. Bury, C. Caputo, P. David, C. Delaere, I. S. Donertas, A. Giammanco, K. Jaffel, Sa. Jain, V. Lemaitre, K. Mondal, J. Prisciandaro, A. Taliercio, M. Teklishyn, T. T. Tran, P. Vischia, S. Wertz, G. A. Alves, C. Hensel, A. Moraes, P. Rebello Teles, W. L. Aldá Júnior, M. Alves Gallo Pereira, M. Barroso Ferreira Filho, H. Brandao Malbouisson, W. Carvalho, J. Chinellato, E. M. Da Costa, G. G. Da Silveira, D. De Jesus Damiao, V. Dos Santos Sousa, S. Fonseca De Souza, C. Mora Herrera, K. Mota Amarilo, L. Mundim, H. Nogima, A. Santoro, S. M. Silva Do Amaral, A. Sznajder, M. Thiel, F. Torres Da Silva De Araujo, A. Vilela Pereira, C. A. Bernardes, L. Calligaris, T. R. Fernandez Perez Tomei, E. M. Gregores, D. S. Lemos, P. G. Mercadante, S. F. Novaes, Sandra S. Padula, A. Aleksandrov, G. Antchev, R. Hadjiiska, P. Iaydjiev, M. Misheva, M. Rodozov, M. Shopova, G. Sultanov, A. Dimitrov, T. Ivanov, L. Litov, B. Pavlov, P. Petkov, A. Petrov, T. Cheng, T. Javaid, M. Mittal, L. Yuan, M. Ahmad, G. Bauer, C. Dozen, Z. Hu, J. Martins, Y. Wang, K. Yi, E. Chapon, G. M. Chen, H. S. Chen, M. Chen, F. Iemmi, A. Kapoor, D. Leggat, H. Liao, Z. -A. Liu, V. Milosevic, F. Monti, R. Sharma, J. Tao, J. Thomas-Wilsker, J. Wang, H. Zhang, J. Zhao, A. Agapitos, Y. An, Y. Ban, C. Chen, A. Levin, Q. Li, X. Lyu, Y. Mao, S. J. Qian, D. Wang, J. Xiao, H. Yang, M. Lu, Z. You, X. Gao, H. Okawa, Y. Zhang, Z. Lin, M. Xiao, C. Avila, A. Cabrera, C. Florez, J. Fraga, J. Mejia Guisao, F. Ramirez, J. D. Ruiz Alvarez, D. Giljanovic, N. Godinovic, D. Lelas, I. Puljak, Z. Antunovic, M. Kovac, T. Sculac, V. Brigljevic, D. Ferencek, D. Majumder, M. Roguljic, A. Starodumov, T. Susa, A. Attikis, K. Christoforou, G. Kole, M. Kolosova, S. Konstantinou, J. Mousa, C. Nicolaou, F. Ptochos, P. A. Razis, H. Rykaczewski, H. Saka, M. Finger, M. Finger, A. Kveton, E. Ayala, E. Carrera Jarrin, A. A. Abdelalim, E. Salama, M. A. Mahmoud, Y. Mohammed, S. Bhowmik, R. K. Dewanjee, K. Ehataht, M. Kadastik, S. Nandan, C. Nielsen, J. Pata, M. Raidal, L. Tani, C. Veelken, P. Eerola, H. Kirschenmann, K. Osterberg, M. Voutilainen, S. Bharthuar, E. Brücken, F. Garcia, J. Havukainen, M. S. Kim, R. Kinnunen, T. Lampén, K. Lassila-Perini, S. Lehti, T. Lindén, M. Lotti, L. Martikainen, M. Myllymäki, J. Ott, M. m. Rantanen, H. Siikonen, E. Tuominen, J. Tuominiemi, P. Luukka, H. Petrow, T. Tuuva, C. Amendola, M. Besancon, F. Couderc, M. Dejardin, D. Denegri, J. L. Faure, F. Ferri, S. Ganjour, P. Gras, G. Hamel de Monchenault, P. Jarry, B. Lenzi, J. Malcles, J. Rander, A. Rosowsky, M. Ö. Sahin, A. Savoy-Navarro, M. Titov, G. B. Yu, S. Ahuja, F. Beaudette, M. Bonanomi, A. Buchot Perraguin, P. Busson, A. Cappati, C. Charlot, O. Davignon, B. Diab, G. Falmagne, S. Ghosh, R. Granier de Cassagnac, A. Hakimi, I. Kucher, J. Motta, M. Nguyen, C. Ochando, P. Paganini, J. Rembser, R. Salerno, U. Sarkar, J. B. Sauvan, Y. Sirois, A. Tarabini, A. Zabi, A. Zghiche, J. -L. Agram, J. Andrea, D. Apparu, D. Bloch, G. Bourgatte, J -M. Brom, E. C. Chabert, C. Collard, D. Darej, J. -C. Fontaine, U. Goerlach, C. Grimault, A. -C. Le Bihan, E. Nibigira, P. Van Hove, E. Asilar, S. Beauceron, C. Bernet, G. Boudoul, C. Camen, A. Carle, N. Chanon, D. Contardo, P. Depasse, H. El Mamouni, J. Fay, S. Gascon, M. Gouzevitch, B. Ille, I. B. Laktineh, H. Lattaud, A. Lesauvage, M. Lethuillier, L. Mirabito, S. Perries, K. Shchablo, V. Sordini, L. Torterotot, G. Touquet, M. Vander Donckt, S. Viret, I. Lomidze, T. Toriashvili, Z. Tsamalaidze, V. Botta, L. Feld, K. Klein, M. Lipinski, D. Meuser, A. Pauls, N. Röwert, J. Schulz, M. Teroerde, A. Dodonova, D. Eliseev, M. Erdmann, P. Fackeldey, B. Fischer, T. Hebbeker, K. Hoepfner, F. Ivone, L. Mastrolorenzo, M. Merschmeyer, A. Meyer, G. Mocellin, S. Mondal, S. Mukherjee, D. Noll, A. Novak, A. Pozdnyakov, Y. Rath, H. Reithler, A. Schmidt, S. C. Schuler, A. Sharma, L. Vigilante, S. Wiedenbeck, S. Zaleski, C. Dziwok, G. Flügge, W. Haj Ahmad, O. Hlushchenko, T. Kress, A. Nowack, O. Pooth, D. Roy, A. Stahl, T. Ziemons, A. Zotz, H. Aarup Petersen, M. Aldaya Martin, P. Asmuss, S. Baxter, M. Bayatmakou, O. Behnke, A. Bermúdez Martínez, S. Bhattacharya, A. A. Bin Anuar, F. Blekman, K. Borras, D. Brunner, A. Campbell, A. Cardini, C. Cheng, F. Colombina, S. Consuegra Rodríguez, G. Correia Silva, M. De Silva, L. Didukh, G. Eckerlin, D. Eckstein, L. I. Estevez Banos, O. Filatov, E. Gallo, A. Geiser, A. Giraldi, A. Grohsjean, M. Guthoff, A. Jafari, N. Z. Jomhari, H. Jung, A. Kasem, M. Kasemann, H. Kaveh, C. Kleinwort, R. Kogler, D. Krücker, W. Lange, K. Lipka, W. Lohmann, R. Mankel, I. -A. Melzer-Pellmann, M. Mendizabal Morentin, J. Metwally, A. B. Meyer, M. Meyer, J. Mnich, A. Mussgiller, A. Nürnberg, Y. Otarid, D. Pérez Adán, D. Pitzl, A. Raspereza, B. Ribeiro Lopes, J. Rübenach, A. Saggio, A. Saibel, M. Savitskyi, M. Scham, V. Scheurer, S. Schnake, P. Schütze, C. Schwanenberger, M. Shchedrolosiev, R. E. Sosa Ricardo, D. Stafford, N. Tonon, M. Van De Klundert, F. Vazzoler, R. Walsh, D. Walter, Q. Wang, Y. Wen, K. Wichmann, L. Wiens, C. Wissing, S. Wuchterl, R. Aggleton, S. Albrecht, S. Bein, L. Benato, P. Connor, K. De Leo, M. Eich, K. El Morabit, F. Feindt, A. Fröhlich, C. Garbers, E. Garutti, P. Gunnellini, M. Hajheidari, J. Haller, A. Hinzmann, G. Kasieczka, R. Klanner, T. Kramer, V. Kutzner, J. Lange, T. Lange, A. Lobanov, A. Malara, C. Matthies, A. Mehta, A. Nigamova, K. J. Pena Rodriguez, M. Rieger, O. Rieger, P. Schleper, M. Schröder, J. Schwandt, J. Sonneveld, H. Stadie, G. Steinbrück, A. Tews, I. Zoi, J. Bechtel, S. Brommer, M. Burkart, E. Butz, R. Caspart, T. Chwalek, W. De Boer, A. Dierlamm, A. Droll, N. Faltermann, M. Giffels, J. O. Gosewisch, A. Gottmann, F. Hartmann, C. Heidecker, U. Husemann, P. Keicher, R. Koppenhöfer, S. Maier, S. Mitra, Th. Müller, M. Neukum, G. Quast, K. Rabbertz, J. Rauser, D. Savoiu, M. Schnepf, D. Seith, I. Shvetsov, H. J. Simonis, R. Ulrich, J. Van Der Linden, R. F. Von Cube, M. Wassmer, M. Weber, S. Wieland, R. Wolf, S. Wozniewski, S. Wunsch, G. Anagnostou, G. Daskalakis, A. Kyriakis, D. Loukas, A. Stakia, M. Diamantopoulou, D. Karasavvas, P. Kontaxakis, C. K. Koraka, A. Manousakis-Katsikakis, A. Panagiotou, I. Papavergou, N. Saoulidou, K. Theofilatos, E. Tziaferi, K. Vellidis, E. Vourliotis, G. Bakas, K. Kousouris, I. Papakrivopoulos, G. Tsipolitis, A. Zacharopoulou, K. Adamidis, I. Bestintzanos, I. Evangelou, C. Foudas, P. Gianneios, P. Katsoulis, P. Kokkas, N. Manthos, I. Papadopoulos, J. Strologas, M. Csanad, K. Farkas, M. M. A. Gadallah, S. Lökös, P. Major, K. Mandal, G. Pasztor, A. J. Rádl, O. Surányi, G. I. Veres, M. Bartók, G. Bencze, C. Hajdu, D. Horvath, F. Sikler, V. Veszpremi, S. Czellar, D. Fasanella, F. Fienga, J. Karancsi, J. Molnar, Z. Szillasi, D. Teyssier, P. Raics, Z. L. Trocsanyi, B. Ujvari, T. Csorgo, F. Nemes, T. Novak, S. Bahinipati, C. Kar, P. Mal, T. Mishra, V. K. Muraleedharan Nair Bindhu, A. Nayak, P. Saha, N. Sur, S. K. Swain, D. Vats, S. Bansal, S. B. Beri, V. Bhatnagar, G. Chaudhary, S. Chauhan, N. Dhingra, R. Gupta, A. Kaur, H. Kaur, M. Kaur, P. Kumari, M. Meena, K. Sandeep, J. B. Singh, A. K. Virdi, A. Ahmed, A. Bhardwaj, B. C. Choudhary, M. Gola, S. Keshri, A. Kumar, M. Naimuddin, P. Priyanka, K. Ranjan, S. Saumya, A. Shah, M. Bharti, R. Bhattacharya, S. Bhattacharya, D. Bhowmik, S. Dutta, S. Dutta, B. Gomber, M. Maity, P. Palit, P. K. Rout, G. Saha, B. Sahu, S. Sarkar, M. Sharan, P. K. Behera, S. C. Behera, P. Kalbhor, J. R. Komaragiri, D. Kumar, A. Muhammad, L. Panwar, R. Pradhan, P. R. Pujahari, A. Sharma, A. K. Sikdar, P. C. Tiwari, K. Naskar, T. Aziz, S. Dugad, M. Kumar, S. Banerjee, R. Chudasama, M. Guchait, S. Karmakar, S. Kumar, G. Majumder, K. Mazumdar, S. Mukherjee, A. Alpana, S. Dube, B. Kansal, A. Laha, S. Pandey, A. Rastogi, S. Sharma, H. Bakhshiansohi, E. Khazaie, M. Zeinali, S. Chenarani, S. M. Etesami, M. Khakzad, M. Mohammadi Najafabadi, M. Grunewald, M. Abbrescia, R. Aly, C. Aruta, A. Colaleo, D. Creanza, N. De Filippis, M. De Palma, A. Di Florio, A. Di Pilato, W. Elmetenawee, F. Errico, L. Fiore, G. Iaselli, M. Ince, S. Lezki, G. Maggi, M. Maggi, I. Margjeka, V. Mastrapasqua, S. My, S. Nuzzo, A. Pellecchia, A. Pompili, G. Pugliese, D. Ramos, A. Ranieri, G. Selvaggi, L. Silvestris, F. M. Simone, Ü. Sözbilir, R. Venditti, P. Verwilligen, G. Abbiendi, C. Battilana, D. Bonacorsi, L. Borgonovi, L. Brigliadori, R. Campanini, P. Capiluppi, A. Castro, F. R. Cavallo, C. Ciocca, M. Cuffiani, G. M. Dallavalle, T. Diotalevi, F. Fabbri, A. Fanfani, P. Giacomelli, L. Giommi, C. Grandi, L. Guiducci, S. Lo Meo, L. Lunerti, S. Marcellini, G. Masetti, F. L. Navarria, A. Perrotta, F. Primavera, A. M. Rossi, T. Rovelli, G. P. Siroli, S. Albergo, S. Costa, A. Di Mattia, R. Potenza, A. Tricomi, C. Tuve, G. Barbagli, A. Cassese, R. Ceccarelli, V. Ciulli, C. Civinini, R. D’Alessandro, E. Focardi, G. Latino, P. Lenzi, M. Lizzo, M. Meschini, S. Paoletti, R. Seidita, G. Sguazzoni, L. Viliani, L. Benussi, S. Bianco, D. Piccolo, M. Bozzo, F. Ferro, R. Mulargia, E. Robutti, S. Tosi, A. Benaglia, G. Boldrini, F. Brivio, F. Cetorelli, F. De Guio, M. E. Dinardo, P. Dini, S. Gennai, A. Ghezzi, P. Govoni, L. Guzzi, M. T. Lucchini, M. Malberti, S. Malvezzi, A. Massironi, D. Menasce, L. Moroni, M. Paganoni, D. Pedrini, B. S. Pinolini, S. Ragazzi, N. Redaelli, T. Tabarelli de Fatis, D. Valsecchi, D. Zuolo, S. Buontempo, F. Carnevali, N. Cavallo, A. De Iorio, F. Fabozzi, A. O. M. Iorio, L. Lista, S. Meola, P. Paolucci, B. Rossi, C. Sciacca, P. Azzi, N. Bacchetta, D. Bisello, P. Bortignon, A. Bragagnolo, R. Carlin, P. Checchia, T. Dorigo, U. Dosselli, F. Gasparini, U. Gasparini, G. Grosso, L. Layer, E. Lusiani, M. Margoni, F. Marini, A. T. Meneguzzo, J. Pazzini, P. Ronchese, R. Rossin, F. Simonetto, G. Strong, M. Tosi, H. Yarar, M. Zanetti, P. Zotto, A. Zucchetta, G. Zumerle, C. Aimè, A. Braghieri, S. Calzaferri, D. Fiorina, P. Montagna, S. P. Ratti, V. Re, C. Riccardi, P. Salvini, I. Vai, P. Vitulo, P. Asenov, G. M. Bilei, D. Ciangottini, L. Fanò, M. Magherini, G. Mantovani, V. Mariani, M. Menichelli, F. Moscatelli, A. Piccinelli, M. Presilla, A. Rossi, A. Santocchia, D. Spiga, T. Tedeschi, P. Azzurri, G. Bagliesi, V. Bertacchi, L. Bianchini, T. Boccali, E. Bossini, R. Castaldi, M. A. Ciocci, V. D’Amante, R. Dell’Orso, M. R. Di Domenico, S. Donato, A. Giassi, F. Ligabue, E. Manca, G. Mandorli, D. Matos Figueiredo, A. Messineo, M. Musich, F. Palla, S. Parolia, G. Ramirez-Sanchez, A. Rizzi, G. Rolandi, S. Roy Chowdhury, A. Scribano, N. Shafiei, P. Spagnolo, R. Tenchini, G. Tonelli, N. Turini, A. Venturi, P. G. Verdini, P. Barria, M. Campana, F. Cavallari, D. Del Re, E. Di Marco, M. Diemoz, E. Longo, P. Meridiani, G. Organtini, F. Pandolfi, R. Paramatti, C. Quaranta, S. Rahatlou, C. Rovelli, F. Santanastasio, L. Soffi, R. Tramontano, N. Amapane, R. Arcidiacono, S. Argiro, M. Arneodo, N. Bartosik, R. Bellan, A. Bellora, J. Berenguer Antequera, C. Biino, N. Cartiglia, M. Costa, R. Covarelli, N. Demaria, M. Grippo, B. Kiani, F. Legger, C. Mariotti, S. Maselli, A. Mecca, E. Migliore, E. Monteil, M. Monteno, M. M. Obertino, G. Ortona, L. Pacher, N. Pastrone, M. Pelliccioni, M. Ruspa, K. Shchelina, F. Siviero, V. Sola, A. Solano, D. Soldi, A. Staiano, M. Tornago, D. Trocino, G. Umoret, A. Vagnerini, S. Belforte, V. Candelise, M. Casarsa, F. Cossutti, A. Da Rold, G. Della Ricca, G. Sorrentino, S. Dogra, C. Huh, B. Kim, D. H. Kim, G. N. Kim, J. Kim, J. Lee, S. W. Lee, C. S. Moon, Y. D. Oh, S. I. Pak, S. Sekmen, Y. C. Yang, H. Kim, D. H. Moon, B. Francois, T. J. Kim, J. Park, S. Cho, S. Choi, B. Hong, K. Lee, K. S. Lee, J. Lim, J. Park, S. K. Park, J. Yoo, J. Goh, A. Gurtu, H. S. Kim, Y. Kim, J. Almond, J. H. Bhyun, J. Choi, S. Jeon, J. Kim, J. S. Kim, S. Ko, H. Kwon, H. Lee, S. Lee, B. H. Oh, M. Oh, S. B. Oh, H. Seo, U. K. Yang, I. Yoon, W. Jang, D. Y. Kang, Y. Kang, S. Kim, B. Ko, J. S. H. Lee, Y. Lee, J. A. Merlin, I. C. Park, Y. Roh, M. S. Ryu, D. Song, I. J. Watson, S. Yang, S. Ha, H. D. Yoo, M. Choi, H. Lee, Y. Lee, I. Yu, T. Beyrouthy, Y. Maghrbi, K. Dreimanis, V. Veckalns, M. Ambrozas, A. Carvalho Antunes De Oliveira, A. Juodagalvis, A. Rinkevicius, G. Tamulaitis, N. Bin Norjoharuddeen, Z. Zolkapli, J. F. Benitez, A. Castaneda Hernandez, H. A. Encinas Acosta, L. G. Gallegos Maríñez, M. León Coello, J. A. Murillo Quijada, A. Sehrawat, L. Valencia Palomo, G. Ayala, H. Castilla-Valdez, E. De La Cruz-Burelo, I. Heredia-De La Cruz, R. Lopez-Fernandez, C. A. Mondragon Herrera, D. A. Perez Navarro, R. Reyes-Almanza, A. Sánchez Hernández, S. Carrillo Moreno, C. Oropeza Barrera, F. Vazquez Valencia, I. Pedraza, H. A. Salazar Ibarguen, C. Uribe Estrada, J. Mijuskovic, N. Raicevic, D. Krofcheck, P. H. Butler, A. Ahmad, M. I. Asghar, A. Awais, M. I. M. Awan, M. Gul, H. R. Hoorani, W. A. Khan, M. A. Shah, M. Shoaib, M. Waqas, V. Avati, L. Grzanka, M. Malawski, H. Bialkowska, M. Bluj, B. Boimska, M. Górski, M. Kazana, M. Szleper, P. Zalewski, K. Bunkowski, K. Doroba, A. Kalinowski, M. Konecki, J. Krolikowski, M. Araujo, P. Bargassa, D. Bastos, A. Boletti, P. Faccioli, M. Gallinaro, J. Hollar, N. Leonardo, T. Niknejad, M. Pisano, J. Seixas, O. Toldaiev, J. Varela, S. Afanasiev, D. Budkouski, I. Golutvin, I. Gorbunov, V. Karjavine, V. Korenkov, A. Lanev, A. Malakhov, V. Matveev, V. Palichik, V. Perelygin, M. Savina, V. Shalaev, S. Shmatov, S. Shulha, V. Smirnov, O. Teryaev, N. Voytishin, B. S. Yuldashev, A. Zarubin, I. Zhizhin, G. Gavrilov, V. Golovtcov, Y. Ivanov, V. Kim, E. Kuznetsova, V. Murzin, V. Oreshkin, I. Smirnov, D. Sosnov, V. Sulimov, L. Uvarov, S. Volkov, A. Vorobyev, Yu. Andreev, A. Dermenev, S. Gninenko, N. Golubev, A. Karneyeu, D. Kirpichnikov, M. Kirsanov, N. Krasnikov, A. Pashenkov, G. Pivovarov, A. Toropin, V. Epshteyn, V. Gavrilov, N. Lychkovskaya, A. Nikitenko, V. Popov, A. Stepennov, M. Toms, E. Vlasov, A. Zhokin, T. Aushev, O. Bychkova, R. Chistov, M. Danilov, A. Oskin, P. Parygin, S. Polikarpov, A. Tulupov, V. Andreev, M. Azarkin, I. Dremin, M. Kirakosyan, A. Terkulov, A. Belyaev, E. Boos, M. Dubinin, L. Dudko, A. Ershov, A. Gribushin, V. Klyukhin, O. Kodolova, I. Lokhtin, S. Obraztsov, S. Petrushanko, V. Savrin, A. Snigirev, V. Blinov, T. Dimova, L. Kardapoltsev, A. Kozyrev, I. Ovtin, O. Radchenko, Y. Skovpen, I. Azhgirey, I. Bayshev, D. Elumakhov, V. Kachanov, D. Konstantinov, P. Mandrik, V. Petrov, R. Ryutin, S. Slabospitskii, A. Sobol, S. Troshin, N. Tyurin, A. Uzunian, A. Volkov, A. Babaev, V. Okhotnikov, V. Borshch, V. Ivanchenko, E. Tcherniaev, P. Adzic, M. Dordevic, P. Milenovic, J. Milosevic, M. Aguilar-Benitez, J. Alcaraz Maestre, A. Álvarez Fernández, I. Bachiller, M. Barrio Luna, Cristina F. Bedoya, C. A. Carrillo Montoya, M. Cepeda, M. Cerrada, N. Colino, B. De La Cruz, A. Delgado Peris, J. P. Fernández Ramos, J. Flix, M. C. Fouz, O. Gonzalez Lopez, S. Goy Lopez, J. M. Hernandez, M. I. Josa, J. León Holgado, D. Moran, Á. Navarro Tobar, C. Perez Dengra, A. Pérez-Calero Yzquierdo, J. Puerta Pelayo, I. Redondo, L. Romero, S. Sánchez Navas, L. Urda Gómez, C. Willmott, J. F. de Trocóniz, B. Alvarez Gonzalez, J. Cuevas, C. Erice, J. Fernandez Menendez, S. Folgueras, I. Gonzalez Caballero, J. R. González Fernández, E. Palencia Cortezon, C. Ramón Álvarez, V. Rodríguez Bouza, A. Soto Rodríguez, A. Trapote, N. Trevisani, C. Vico Villalba, J. A. Brochero Cifuentes, I. J. Cabrillo, A. Calderon, J. Duarte Campderros, M. Fernandez, C. Fernandez Madrazo, P. J. Fernández Manteca, A. García Alonso, G. Gomez, C. Martinez Rivero, P. Martinez Ruiz del Arbol, F. Matorras, P. Matorras Cuevas, J. Piedra Gomez, C. Prieels, A. Ruiz-Jimeno, L. Scodellaro, I. Vila, J. M. Vizan Garcia, M. K. Jayananda, B. Kailasapathy, D. U. J. Sonnadara, D. D. C. Wickramarathna, W. G. D. Dharmaratna, K. Liyanage, N. Perera, N. Wickramage, T. K. Aarrestad, D. Abbaneo, J. Alimena, E. Auffray, G. Auzinger, J. Baechler, P. Baillon, D. Barney, J. Bendavid, M. Bianco, A. Bocci, C. Caillol, T. Camporesi, M. Capeans Garrido, G. Cerminara, N. Chernyavskaya, S. S. Chhibra, S. Choudhury, M. Cipriani, L. Cristella, D. d’Enterria, A. Dabrowski, A. David, A. De Roeck, M. M. Defranchis, M. Deile, M. Dobson, M. Dünser, N. Dupont, A. Elliott-Peisert, F. Fallavollita, A. Florent, L. Forthomme, G. Franzoni, W. Funk, S. Ghosh, S. Giani, D. Gigi, K. Gill, F. Glege, L. Gouskos, E. Govorkova, M. Haranko, J. Hegeman, V. Innocente, T. James, P. Janot, J. Kaspar, J. Kieseler, M. Komm, N. Kratochwil, C. Lange, S. Laurila, P. Lecoq, A. Lintuluoto, K. Long, C. Lourenço, B. Maier, L. Malgeri, S. Mallios, M. Mannelli, A. C. Marini, F. Meijers, S. Mersi, E. Meschi, F. Moortgat, M. Mulders, S. Orfanelli, L. Orsini, F. Pantaleo, E. Perez, M. Peruzzi, A. Petrilli, G. Petrucciani, A. Pfeiffer, M. Pierini, D. Piparo, M. Pitt, H. Qu, T. Quast, D. Rabady, A. Racz, G. Reales Gutiérrez, M. Rovere, H. Sakulin, J. Salfeld-Nebgen, S. Scarfi, C. Schwick, M. Selvaggi, A. Sharma, P. Silva, W. Snoeys, P. Sphicas, S. Summers, K. Tatar, V. R. Tavolaro, D. Treille, P. Tropea, A. Tsirou, J. Wanczyk, K. A. Wozniak, W. D. Zeuner, L. Caminada, A. Ebrahimi, W. Erdmann, R. Horisberger, Q. Ingram, H. C. Kaestli, D. Kotlinski, U. Langenegger, M. Missiroli, L. Noehte, T. Rohe, K. Androsov, M. Backhaus, P. Berger, A. Calandri, A. De Cosa, G. Dissertori, M. Dittmar, M. Donegà, C. Dorfer, F. Eble, K. Gedia, F. Glessgen, T. A. Gómez Espinosa, C. Grab, D. Hits, W. Lustermann, A. -M. Lyon, R. A. Manzoni, L. Marchese, C. Martin Perez, M. T. Meinhard, F. Nessi-Tedaldi, J. Niedziela, F. Pauss, V. Perovic, S. Pigazzini, M. G. Ratti, M. Reichmann, C. Reissel, T. Reitenspiess, B. Ristic, D. Ruini, D. A. Sanz Becerra, V. Stampf, J. Steggemann, R. Wallny, C. Amsler, P. Bärtschi, C. Botta, D. Brzhechko, M. F. Canelli, K. Cormier, A. De Wit, R. Del Burgo, J. K. Heikkilä, M. Huwiler, W. Jin, A. Jofrehei, B. Kilminster, S. Leontsinis, S. P. Liechti, A. Macchiolo, P. Meiring, V. M. Mikuni, U. Molinatti, I. Neutelings, A. Reimers, P. Robmann, S. Sanchez Cruz, K. Schweiger, M. Senger, Y. Takahashi, C. Adloff, C. M. Kuo, W. Lin, A. Roy, T. Sarkar, S. S. Yu, L. Ceard, Y. Chao, K. F. Chen, P. H. Chen, P. s. Chen, H. Cheng, W. -S. Hou, Y. y. Li, R. -S. Lu, E. Paganis, A. Psallidas, A. Steen, H. y. Wu, E. Yazgan, P. r. Yu, B. Asavapibhop, C. Asawatangtrakuldee, N. Srimanobhas, F. Boran, S. Damarseckin, Z. S. Demiroglu, F. Dolek, I. Dumanoglu, E. Eskut, Y. Guler, E. Gurpinar Guler, C. Isik, O. Kara, A. Kayis Topaksu, U. Kiminsu, G. Onengut, K. Ozdemir, A. Polatoz, A. E. Simsek, B. Tali, U. G. Tok, S. Turkcapar, I. S. Zorbakir, G. Karapinar, K. Ocalan, M. Yalvac, B. Akgun, I. O. Atakisi, E. Gulmez, M. Kaya, O. Kaya, Ö. Özçelik, S. Tekten, E. A. Yetkin, A. Cakir, K. Cankocak, Y. Komurcu, S. Sen, S. Cerci, I. Hos, B. Isildak, B. Kaynak, S. Ozkorucuklu, H. Sert, C. Simsek, D. Sunar Cerci, C. Zorbilmez, B. Grynyov, L. Levchuk, D. Anthony, E. Bhal, S. Bologna, J. J. Brooke, A. Bundock, E. Clement, D. Cussans, H. Flacher, M. Glowacki, J. Goldstein, G. P. Heath, H. F. Heath, L. Kreczko, B. Krikler, S. Paramesvaran, S. Seif El Nasr-Storey, V. J. Smith, N. Stylianou, K. Walkingshaw Pass, R. White, K. W. Bell, A. Belyaev, C. Brew, R. M. Brown, D. J. A. Cockerill, C. Cooke, K. V. Ellis, K. Harder, S. Harper, M. -L. Holmberg, J. Linacre, K. Manolopoulos, D. M. Newbold, E. Olaiya, D. Petyt, T. Reis, T. Schuh, C. H. Shepherd-Themistocleous, I. R. Tomalin, T. Williams, R. Bainbridge, P. Bloch, S. Bonomally, J. Borg, S. Breeze, O. Buchmuller, V. Cepaitis, G. S. Chahal, D. Colling, P. Dauncey, G. Davies, M. Della Negra, S. Fayer, G. Fedi, G. Hall, M. H. Hassanshahi, G. Iles, J. Langford, L. Lyons, A. -M. Magnan, S. Malik, A. Martelli, D. G. Monk, J. Nash, M. Pesaresi, B. C. Radburn-Smith, D. M. Raymond, A. Richards, A. Rose, E. Scott, C. Seez, A. Shtipliyski, A. Tapper, K. Uchida, T. Virdee, M. Vojinovic, N. Wardle, S. N. Webb, D. Winterbottom, K. Coldham, J. E. Cole, A. Khan, P. Kyberd, I. D. Reid, L. Teodorescu, S. Zahid, S. Abdullin, A. Brinkerhoff, B. Caraway, J. Dittmann, K. Hatakeyama, A. R. Kanuganti, B. McMaster, M. Saunders, S. Sawant, C. Sutantawibul, J. Wilson, R. Bartek, A. Dominguez, R. Uniyal, A. M. Vargas Hernandez, A. Buccilli, S. I. Cooper, D. Di Croce, S. V. Gleyzer, C. Henderson, C. U. Perez, P. Rumerio, C. West, A. Akpinar, A. Albert, D. Arcaro, C. Cosby, Z. Demiragli, E. Fontanesi, D. Gastler, S. May, J. Rohlf, K. Salyer, D. Sperka, D. Spitzbart, I. Suarez, A. Tsatsos, S. Yuan, D. Zou, G. Benelli, B. Burkle, X. Coubez, D. Cutts, M. Hadley, U. Heintz, J. M. Hogan, T. Kwon, G. Landsberg, K. T. Lau, D. Li, M. Lukasik, J. Luo, M. Narain, N. Pervan, S. Sagir, F. Simpson, E. Usai, W. Y. Wong, X. Yan, D. Yu, W. Zhang, J. Bonilla, C. Brainerd, R. Breedon, M. Calderon De La Barca Sanchez, M. Chertok, J. Conway, P. T. Cox, R. Erbacher, G. Haza, F. Jensen, O. Kukral, R. Lander, M. Mulhearn, D. Pellett, B. Regnery, D. Taylor, Y. Yao, F. Zhang, M. Bachtis, R. Cousins, A. Datta, D. Hamilton, J. Hauser, M. Ignatenko, M. A. Iqbal, T. Lam, W. A. Nash, S. Regnard, D. Saltzberg, B. Stone, V. Valuev, Y. Chen, R. Clare, J. W. Gary, M. Gordon, G. Hanson, G. Karapostoli, O. R. Long, N. Manganelli, W. Si, S. Wimpenny, Y. Zhang, J. G. Branson, P. Chang, S. Cittolin, S. Cooperstein, D. Diaz, J. Duarte, R. Gerosa, L. Giannini, J. Guiang, R. Kansal, V. Krutelyov, R. Lee, J. Letts, M. Masciovecchio, F. Mokhtar, M. Pieri, B. V. Sathia Narayanan, V. Sharma, M. Tadel, F. Würthwein, Y. Xiang, A. Yagil, N. Amin, C. Campagnari, M. Citron, G. Collura, A. Dorsett, V. Dutta, J. Incandela, M. Kilpatrick, J. Kim, B. Marsh, H. Mei, M. Oshiro, M. Quinnan, J. Richman, U. Sarica, F. Setti, J. Sheplock, P. Siddireddy, D. Stuart, S. Wang, A. Bornheim, O. Cerri, I. Dutta, J. M. Lawhorn, N. Lu, J. Mao, H. B. Newman, T. Q. Nguyen, M. Spiropulu, J. R. Vlimant, C. Wang, S. Xie, Z. Zhang, R. Y. Zhu, J. Alison, S. An, M. B. Andrews, P. Bryant, T. Ferguson, A. Harilal, C. Liu, T. Mudholkar, M. Paulini, A. Sanchez, W. Terrill, J. P. Cumalat, W. T. Ford, A. Hassani, G. Karathanasis, E. MacDonald, R. Patel, A. Perloff, C. Savard, N. Schonbeck, K. Stenson, K. A. Ulmer, S. R. Wagner, N. Zipper, J. Alexander, S. Bright-Thonney, X. Chen, Y. Cheng, D. J. Cranshaw, S. Hogan, J. Monroy, J. R. Patterson, D. Quach, J. Reichert, M. Reid, A. Ryd, W. Sun, J. Thom, P. Wittich, R. Zou, M. Albrow, M. Alyari, G. Apollinari, A. Apresyan, A. Apyan, L. A. T. Bauerdick, D. Berry, J. Berryhill, P. C. Bhat, K. Burkett, J. N. Butler, A. Canepa, G. B. Cerati, H. W. K. Cheung, F. Chlebana, K. F. Di Petrillo, J. Dickinson, V. D. Elvira, Y. Feng, J. Freeman, Z. Gecse, L. Gray, D. Green, S. Grünendahl, O. Gutsche, R. M. Harris, R. Heller, T. C. Herwig, J. Hirschauer, B. Jayatilaka, S. Jindariani, M. Johnson, U. Joshi, T. Klijnsma, B. Klima, K. H. M. Kwok, S. Lammel, D. Lincoln, R. Lipton, T. Liu, C. Madrid, K. Maeshima, C. Mantilla, D. Mason, P. McBride, P. Merkel, S. Mrenna, S. Nahn, J. Ngadiuba, V. Papadimitriou, N. Pastika, K. Pedro, C. Pena, F. Ravera, A. Reinsvold Hall, L. Ristori, E. Sexton-Kennedy, N. Smith, A. Soha, L. Spiegel, S. Stoynev, J. Strait, L. Taylor, S. Tkaczyk, N. V. Tran, L. Uplegger, E. W. Vaandering, H. A. Weber, P. Avery, D. Bourilkov, L. Cadamuro, V. Cherepanov, R. D. Field, D. Guerrero, M. Kim, E. Koenig, J. Konigsberg, A. Korytov, K. H. Lo, K. Matchev, N. Menendez, G. Mitselmakher, A. Muthirakalayil Madhu, N. Rawal, D. Rosenzweig, S. Rosenzweig, K. Shi, J. Wang, Z. Wu, E. Yigitbasi, X. Zuo, T. Adams, A. Askew, R. Habibullah, V. Hagopian, K. F. Johnson, R. Khurana, T. Kolberg, G. Martinez, H. Prosper, C. Schiber, O. Viazlo, R. Yohay, J. Zhang, M. M. Baarmand, S. Butalla, T. Elkafrawy, M. Hohlmann, R. Kumar Verma, D. Noonan, M. Rahmani, F. Yumiceva, M. R. Adams, H. Becerril Gonzalez, R. Cavanaugh, S. Dittmer, O. Evdokimov, C. E. Gerber, D. J. Hofman, A. H. Merrit, C. Mills, G. Oh, T. Roy, S. Rudrabhatla, M. B. Tonjes, N. Varelas, J. Viinikainen, X. Wang, Z. Ye, M. Alhusseini, K. Dilsiz, L. Emediato, R. P. Gandrajula, O. K. Köseyan, J. -P. Merlo, A. Mestvirishvili, J. Nachtman, H. Ogul, Y. Onel, A. Penzo, C. Snyder, E. Tiras, O. Amram, B. Blumenfeld, L. Corcodilos, J. Davis, A. V. Gritsan, S. Kyriacou, P. Maksimovic, J. Roskes, M. Swartz, T.Á. Vámi, A. Abreu, J. Anguiano, C. Baldenegro Barrera, P. Baringer, A. Bean, Z. Flowers, T. Isidori, S. Khalil, J. King, G. Krintiras, A. Kropivnitskaya, M. Lazarovits, C. Le Mahieu, C. Lindsey, J. Marquez, N. Minafra, M. Murray, M. Nickel, C. Rogan, C. Royon, R. Salvatico, S. Sanders, E. Schmitz, C. Smith, Q. Wang, Z. Warner, J. Williams, G. Wilson, S. Duric, A. Ivanov, K. Kaadze, D. Kim, Y. Maravin, T. Mitchell, A. Modak, K. Nam, F. Rebassoo, D. Wright, E. Adams, A. Baden, O. Baron, A. Belloni, S. C. Eno, N. J. Hadley, S. Jabeen, R. G. Kellogg, T. Koeth, Y. Lai, S. Lascio, A. C. Mignerey, S. Nabili, C. Palmer, M. Seidel, A. Skuja, L. Wang, K. Wong, D. Abercrombie, G. Andreassi, R. Bi, W. Busza, I. A. Cali, Y. Chen, M. D’Alfonso, J. Eysermans, C. Freer, G. Gomez Ceballos, M. Goncharov, P. Harris, M. Hu, M. Klute, D. Kovalskyi, J. Krupa, Y. -J. Lee, C. Mironov, C. Paus, D. Rankin, C. Roland, G. Roland, Z. Shi, G. S. F. Stephans, J. Wang, Z. Wang, B. Wyslouch, R. M. Chatterjee, A. Evans, J. Hiltbrand, Sh. Jain, B. M. Joshi, M. Krohn, Y. Kubota, J. Mans, M. Revering, R. Rusack, R. Saradhy, N. Schroeder, N. Strobbe, M. A. Wadud, K. Bloom, M. Bryson, S. Chauhan, D. R. Claes, C. Fangmeier, L. Finco, F. Golf, C. Joo, I. Kravchenko, I. Reed, J. E. Siado, G. R. Snow, W. Tabb, A. Wightman, F. Yan, A. G. Zecchinelli, G. Agarwal, H. Bandyopadhyay, L. Hay, I. Iashvili, A. Kharchilava, C. McLean, D. Nguyen, J. Pekkanen, S. Rappoccio, A. Williams, G. Alverson, E. Barberis, Y. Haddad, Y. Han, A. Hortiangtham, A. Krishna, J. Li, J. Lidrych, G. Madigan, B. Marzocchi, D. M. Morse, V. Nguyen, T. Orimoto, A. Parker, L. Skinnari, A. Tishelman-Charny, T. Wamorkar, B. Wang, A. Wisecarver, D. Wood, S. Bhattacharya, J. Bueghly, Z. Chen, A. Gilbert, T. Gunter, K. A. Hahn, Y. Liu, N. Odell, M. H. Schmitt, M. Velasco, R. Band, R. Bucci, M. Cremonesi, A. Das, N. Dev, R. Goldouzian, M. Hildreth, K. Hurtado Anampa, C. Jessop, K. Lannon, J. Lawrence, N. Loukas, D. Lutton, J. Mariano, N. Marinelli, I. Mcalister, T. McCauley, C. Mcgrady, K. Mohrman, C. Moore, Y. Musienko, R. Ruchti, A. Townsend, M. Wayne, M. Zarucki, L. Zygala, B. Bylsma, L. S. Durkin, B. Francis, C. Hill, M. Nunez Ornelas, K. Wei, B. L. Winer, B. R. Yates, F. M. Addesa, B. Bonham, P. Das, G. Dezoort, P. Elmer, A. Frankenthal, B. Greenberg, N. Haubrich, S. Higginbotham, A. Kalogeropoulos, G. Kopp, S. Kwan, D. Lange, D. Marlow, K. Mei, I. Ojalvo, J. Olsen, D. Stickland, C. Tully, S. Malik, S. Norberg, A. S. Bakshi, V. E. Barnes, R. Chawla, S. Das, L. Gutay, M. Jones, A. W. Jung, D. Kondratyev, A. M. Koshy, M. Liu, G. Negro, N. Neumeister, G. Paspalaki, S. Piperov, A. Purohit, J. F. Schulte, M. Stojanovic, J. Thieman, F. Wang, R. Xiao, W. Xie, J. Dolen, N. Parashar, D. Acosta, A. Baty, T. Carnahan, M. Decaro, S. Dildick, K. M. Ecklund, S. Freed, P. Gardner, F. J. M. Geurts, A. Kumar, W. Li, B. P. Padley, R. Redjimi, J. Rotter, W. Shi, A. G. Stahl Leiton, S. Yang, L. Zhang, Y. Zhang, A. Bodek, P. de Barbaro, R. Demina, J. L. Dulemba, C. Fallon, T. Ferbel, M. Galanti, A. Garcia-Bellido, O. Hindrichs, A. Khukhunaishvili, E. Ranken, R. Taus, G. P. Van Onsem, K. Goulianos, B. Chiarito, J. P. Chou, A. Gandrakota, Y. Gershtein, E. Halkiadakis, A. Hart, M. Heindl, O. Karacheban, I. Laflotte, A. Lath, R. Montalvo, K. Nash, M. Osherson, S. Salur, S. Schnetzer, S. Somalwar, R. Stone, S. A. Thayil, S. Thomas, H. Wang, H. Acharya, A. G. Delannoy, S. Fiorendi, S. Spanier, O. Bouhali, M. Dalchenko, A. Delgado, R. Eusebi, J. Gilmore, T. Huang, T. Kamon, H. Kim, S. Luo, S. Malhotra, R. Mueller, D. Overton, D. Rathjens, A. Safonov, N. Akchurin, J. Damgov, V. Hegde, K. Lamichhane, S. W. Lee, T. Mengke, S. Muthumuni, T. Peltola, I. Volobouev, Z. Wang, A. Whitbeck, E. Appelt, S. Greene, A. Gurrola, W. Johns, A. Melo, K. Padeken, F. Romeo, P. Sheldon, S. Tuo, J. Velkovska, M. W. Arenton, B. Cardwell, B. Cox, G. Cummings, J. Hakala, R. Hirosky, M. Joyce, A. Ledovskoy, A. Li, C. Neu, C. E. Perez Lara, B. Tannenwald, S. White, N. Poudyal, S. Banerjee, K. Black, T. Bose, S. Dasu, I. De Bruyn, P. Everaerts, C. Galloni, H. He, M. Herndon, A. Herve, U. Hussain, A. Lanaro, A. Loeliger, R. Loveless, J. Madhusudanan Sreekala, A. Mallampalli, A. Mohammadi, D. Pinna, A. Savin, V. Shang, V. Sharma, W. H. Smith, D. Teague, S. Trembath-Reichert, W. Vetens, CMS Collaboration

**Affiliations:** 1 Yerevan Physics Institute, Yerevan, Armenia; 2 Institut für Hochenergiephysik, Vienna, Austria; 3 Institute for Nuclear Problems, Minsk, Belarus; 4 Universiteit Antwerpen, Antwerpen, Belgium; 5 Vrije Universiteit Brussel, Brussels, Belgium; 6 Université Libre de Bruxelles, Brussels, Belgium; 7 Ghent University, Ghent, Belgium; 8 Université Catholique de Louvain, Louvain-la-Neuve, Belgium; 9 Centro Brasileiro de Pesquisas Fisicas, Rio de Janeiro, Brazil; 10 Universidade do Estado do Rio de Janeiro, Rio de Janeiro, Brazil; 11 Universidade Estadual Paulista (a), Universidade Federal do ABC (b), São Paulo, Brazil; 12 Institute for Nuclear Research and Nuclear Energy, Bulgarian Academy of Sciences, Sofia, Bulgaria; 13 University of Sofia, Sofia, Bulgaria; 14 Beihang University, Beijing, China; 15 Department of Physics, Tsinghua University, Beijing, China; 16 Institute of High Energy Physics, Beijing, China; 17 State Key Laboratory of Nuclear Physics and Technology, Peking University, Beijing, China; 18 Sun Yat-Sen University, Guangzhou, China; 19 Institute of Modern Physics and Key Laboratory of Nuclear Physics and Ion-beam Application (MOE), Fudan University, Shanghai, China; 20 Zhejiang University, Hangzhou, China, Zhejiang, China; 21 Universidad de Los Andes, Bogota, Colombia; 22 Universidad de Antioquia, Medellin, Colombia; 23 University of Split, Faculty of Electrical Engineering, Mechanical Engineering and Naval Architecture, Split, Croatia; 24 University of Split, Faculty of Science, Split, Croatia; 25 Institute Rudjer Boskovic, Zagreb, Croatia; 26 University of Cyprus, Nicosia, Cyprus; 27 Charles University, Prague, Czech Republic; 28 Escuela Politecnica Nacional, Quito, Ecuador; 29 Universidad San Francisco de Quito, Quito, Ecuador; 30 Academy of Scientific Research and Technology of the Arab Republic of Egypt, Egyptian Network of High Energy Physics, Cairo, Egypt; 31 Center for High Energy Physics (CHEP-FU), Fayoum University, El-Fayoum, Egypt; 32 National Institute of Chemical Physics and Biophysics, Tallinn, Estonia; 33 Department of Physics, University of Helsinki, Helsinki, Finland; 34 Helsinki Institute of Physics, Helsinki, Finland; 35 Lappeenranta University of Technology, Lappeenranta, Finland; 36 IRFU, CEA, Université Paris-Saclay, Gif-sur-Yvette, France; 37 Laboratoire Leprince-Ringuet, CNRS/IN2P3, Ecole Polytechnique, Institut Polytechnique de Paris, Palaiseau, France; 38 Université de Strasbourg, CNRS, IPHC UMR 7178, Strasbourg, France; 39 Institut de Physique des 2 Infinis de Lyon (IP2I), Villeurbanne, France; 40 Georgian Technical University, Tbilisi, Georgia; 41 RWTH Aachen University, I. Physikalisches Institut, Aachen, Germany; 42 RWTH Aachen University, III. Physikalisches Institut A, Aachen, Germany; 43 RWTH Aachen University, III. Physikalisches Institut B, Aachen, Germany; 44 Deutsches Elektronen-Synchrotron, Hamburg, Germany; 45 University of Hamburg, Hamburg, Germany; 46 Karlsruher Institut fuer Technologie, Karlsruhe, Germany; 47 Institute of Nuclear and Particle Physics (INPP), NCSR Demokritos, Aghia Paraskevi, Greece; 48 National and Kapodistrian University of Athens, Athens, Greece; 49 National Technical University of Athens, Athens, Greece; 50 University of Ioánnina, Ioánnina, Greece; 51 MTA-ELTE Lendület CMS Particle and Nuclear Physics Group, Eötvös Loránd University, Budapest, Hungary; 52 Wigner Research Centre for Physics, Budapest, Hungary; 53 Institute of Nuclear Research ATOMKI, Debrecen, Hungary; 54 Institute of Physics, University of Debrecen, Debrecen, Hungary; 55 Karoly Robert Campus, MATE Institute of Technology, Gyongyos, Hungary; 56 National Institute of Science Education and Research, HBNI, Bhubaneswar, India; 57 Panjab University, Chandigarh, India; 58 University of Delhi, Delhi, India; 59 Saha Institute of Nuclear Physics, HBNI, Kolkata, India; 60 Indian Institute of Technology Madras, Madras, India; 61 Bhabha Atomic Research Centre, Mumbai, India; 62 Tata Institute of Fundamental Research-A, Mumbai, India; 63 Tata Institute of Fundamental Research-B, Mumbai, India; 64 Indian Institute of Science Education and Research (IISER), Pune, India; 65 Isfahan University of Technology, Isfahan, Iran; 66 Institute for Research in Fundamental Sciences (IPM), Tehran, Iran; 67 University College Dublin, Dublin, Ireland; 68 INFN Sezione di Bari, Università di Bari, Politecnico di Bari, Bari, Italy; 69 INFN Sezione di Bologna, Università di Bologna, Bologna, Italy; 70 INFN Sezione di Catania, Università di Catania, Catania, Italy; 71 INFN Sezione di Firenze, Università di Firenze, Firenze, Italy; 72 INFN Laboratori Nazionali di Frascati, Frascati, Italy; 73 INFN Sezione di Genova, Università di Genova, Genoa, Italy; 74 INFN Sezione di Milano-Bicocca, Università di Milano-Bicocca, Milan, Italy; 75 INFN Sezione di Napoli, Università di Napoli ‘Federico II’, Napoli, Italy, Università della Basilicata, Potenza, Italy, Università G. Marconi, Rome, Italy; 76 INFN Sezione di Padova, Università di Padova, Padua, Italy, Università di Trento, Trento, Italy; 77 INFN Sezione di Pavia, Università di Pavia, Pavia, Italy; 78 INFN Sezione di Perugia, Università di Perugia, Perugia, Italy; 79 INFN Sezione di Pisa, Università di Pisa, Scuola Normale Superiore di Pisa, Pisa, Italy, Università di Siena, Siena, Italy; 80 INFN Sezione di Roma, Sapienza Università di Roma, Rome, Italy; 81 INFN Sezione di Torino, Università di Torino, Turin, Italy, Università del Piemonte Orientale, Novara, Italy; 82 INFN Sezione di Trieste, Università di Trieste, Trieste, Italy; 83 Kyungpook National University, Daegu, Korea; 84 Chonnam National University, Institute for Universe and Elementary Particles, Kwangju, Korea; 85 Hanyang University, Seoul, Korea; 86 Korea University, Seoul, Korea; 87 Department of Physics, Kyung Hee University, Seoul, Republic of Korea; 88 Sejong University, Seoul, Korea; 89 Seoul National University, Seoul, Korea; 90 University of Seoul, Seoul, Korea; 91 Department of Physics, Yonsei University, Seoul, Korea; 92 Sungkyunkwan University, Suwon, Korea; 93 College of Engineering and Technology, American University of the Middle East (AUM), Egaila, Kuwait, Dasman, Kuwait; 94 Riga Technical University, Riga, Latvia; 95 Vilnius University, Vilnius, Lithuania; 96 National Centre for Particle Physics, Universiti Malaya, Kuala Lumpur, Malaysia; 97 Universidad de Sonora (UNISON), Hermosillo, Mexico; 98 Centro de Investigacion y de Estudios Avanzados del IPN, Mexico City, Mexico; 99 Universidad Iberoamericana, Mexico City, Mexico; 100 Benemerita Universidad Autonoma de Puebla, Puebla, Mexico; 101 University of Montenegro, Podgorica, Montenegro; 102 University of Auckland, Auckland, New Zealand; 103 University of Canterbury, Christchurch, New Zealand; 104 National Centre for Physics, Quaid-I-Azam University, Islamabad, Pakistan; 105 AGH University of Science and Technology Faculty of Computer Science, Electronics and Telecommunications, Krakow, Poland; 106 National Centre for Nuclear Research, Swierk, Poland; 107 Institute of Experimental Physics, Faculty of Physics, University of Warsaw, Warsaw, Poland; 108 Laboratório de Instrumentaç ao e Física Experimental de Partículas, Lisbon, Portugal; 109 Joint Institute for Nuclear Research, Dubna, Russia; 110 Petersburg Nuclear Physics Institute, Gatchina (St. Petersburg), Russia; 111 Institute for Nuclear Research, Moscow, Russia; 112 Institute for Theoretical and Experimental Physics named by A.I. Alikhanov of NRC ‘Kurchatov Institute’, Moscow, Russia; 113 Moscow Institute of Physics and Technology, Moscow, Russia; 114 National Research Nuclear University ‘Moscow Engineering Physics Institute’ (MEPhI), Moscow, Russia; 115 P.N. Lebedev Physical Institute, Moscow, Russia; 116 Skobeltsyn Institute of Nuclear Physics, Lomonosov Moscow State University, Moscow, Russia; 117 Novosibirsk State University (NSU), Novosibirsk, Russia; 118 Institute for High Energy Physics of National Research Centre ‘Kurchatov Institute’, Protvino, Russia; 119 National Research Tomsk Polytechnic University, Tomsk, Russia; 120 Tomsk State University, Tomsk, Russia; 121 University of Belgrade: Faculty of Physics and VINCA Institute of Nuclear Sciences, Belgrade, Serbia; 122 Centro de Investigaciones Energéticas Medioambientales y Tecnológicas (CIEMAT), Madrid, Spain; 123 Universidad Autónoma de Madrid, Madrid, Spain; 124 Universidad de Oviedo, Instituto Universitario de Ciencias y Tecnologías Espaciales de Asturias (ICTEA), Oviedo, Spain; 125 Instituto de Física de Cantabria (IFCA), CSIC-Universidad de Cantabria, Santander, Spain; 126 University of Colombo, Colombo, Sri Lanka; 127 Department of Physics, University of Ruhuna, Matara, Sri Lanka; 128 CERN, European Organization for Nuclear Research, Geneva, Switzerland; 129 Paul Scherrer Institut, Villigen, Switzerland; 130 ETH Zurich-Institute for Particle Physics and Astrophysics (IPA), Zurich, Switzerland; 131 Universität Zürich, Zurich, Switzerland; 132 National Central University, Chung-Li, Taiwan; 133 National Taiwan University (NTU), Taipei, Taiwan; 134 Department of Physics, Faculty of Science, Chulalongkorn University, Bangkok, Thailand; 135 Physics Department, Science and Art Faculty, Çukurova University, Adana, Turkey; 136 Physics Department, Middle East Technical University, Ankara, Turkey; 137 Bogazici University, Istanbul, Turkey; 138 Istanbul Technical University, Istanbul, Turkey; 139 Istanbul University, Istanbul, Turkey; 140 Institute for Scintillation Materials of National Academy of Science of Ukraine, Kharkov, Ukraine; 141 National Scientific Center, Kharkov Institute of Physics and Technology, Kharkov, Ukraine; 142 University of Bristol, Bristol, UK; 143 Rutherford Appleton Laboratory, Didcot, UK; 144 Imperial College, London, UK; 145 Brunel University, Uxbridge, UK; 146 Baylor University, Waco, TX USA; 147 Catholic University of America, Washington, DC USA; 148 The University of Alabama, Tuscaloosa, AL USA; 149 Boston University, Boston, MA USA; 150 Brown University, Providence, RI USA; 151 University of California, Davis, Davis, CA USA; 152 University of California, Los Angeles, CA USA; 153 University of California, Riverside, Riverside, CA USA; 154 University of California, San Diego, La Jolla, CA USA; 155 Department of Physics, University of California, Santa Barbara, Santa Barbara, CA USA; 156 California Institute of Technology, Pasadena, CA USA; 157 Carnegie Mellon University, Pittsburgh, PA USA; 158 University of Colorado Boulder, Boulder, CO USA; 159 Cornell University, Ithaca, NY USA; 160 Fermi National Accelerator Laboratory, Batavia, IL USA; 161 University of Florida, Gainesville, FL USA; 162 Florida State University, Tallahassee, FL USA; 163 Florida Institute of Technology, Melbourne, FL USA; 164 University of Illinois at Chicago (UIC), Chicago, IL USA; 165 The University of Iowa, Iowa City, IA USA; 166 Johns Hopkins University, Baltimore, MD USA; 167 The University of Kansas, Lawrence, KS USA; 168 Kansas State University, Manhattan, KS USA; 169 Lawrence Livermore National Laboratory, Livermore, CA USA; 170 University of Maryland, College Park, MD USA; 171 Massachusetts Institute of Technology, Cambridge, MA USA; 172 University of Minnesota, Minneapolis, MN USA; 173 University of Nebraska-Lincoln, Lincoln, NE USA; 174 State University of New York at Buffalo, Buffalo, NY USA; 175 Northeastern University, Boston, MA USA; 176 Northwestern University, Evanston, IL USA; 177 University of Notre Dame, Notre Dame, IN USA; 178 The Ohio State University, Columbus, OH USA; 179 Princeton University, Princeton, NJ USA; 180 University of Puerto Rico, Mayaguez, PR USA; 181 Purdue University, West Lafayette, IN USA; 182 Purdue University Northwest, Hammond, IN USA; 183 Rice University, Houston, TX USA; 184 University of Rochester, Rochester, NY USA; 185 The Rockefeller University, New York, NY USA; 186 Rutgers, The State University of New Jersey, Piscataway, NJ USA; 187 University of Tennessee, Knoxville, TN USA; 188 Texas A &M University, College Station, TX USA; 189 Texas Tech University, Lubbock, TX USA; 190 Vanderbilt University, Nashville, TN USA; 191 University of Virginia, Charlottesville, VA USA; 192 Wayne State University, Detroit, MI USA; 193 University of Wisconsin-Madison, Madison, WI USA; 194 TU Wien, Vienna, Austria; 195 Institute of Basic and Applied Sciences, Faculty of Engineering, Arab Academy for Science, Technology and Maritime Transport, Alexandria, Egypt; 196 Université Libre de Bruxelles, Brussels, Belgium; 197 Universidade Estadual de Campinas, Campinas, Brazil; 198 Federal University of Rio Grande do Sul, Porto Alegre, Brazil; 199 The University of the State of Amazonas, Manaus, Brazil; 200 University of Chinese Academy of Sciences, Beijing, China; 201 Department of Physics, Tsinghua University, Beijing, China; 202 UFMS, Nova Andradina, Brazil; 203 Department of Physics, Nanjing Normal University, Nanjing, China; 204 The University of Iowa, Iowa City, IA USA; 205 Institute for Theoretical and Experimental Physics named by A.I. Alikhanov of NRC ‘Kurchatov Institute’, Moscow, Russia; 206 Joint Institute for Nuclear Research, Dubna, Russia; 207 Helwan University, Cairo, Egypt; 208 Zewail City of Science and Technology, Zewail, Egypt; 209 British University in Egypt, Cairo, Egypt; 210 Ain Shams University, Cairo, Egypt; 211 Purdue University, West Lafayette, IN USA; 212 Université de Haute Alsace, Mulhouse, France; 213 Tbilisi State University, Tbilisi, Georgia; 214 Erzincan Binali Yildirim University, Erzincan, Turkey; 215 CERN, European Organization for Nuclear Research, Geneva, Switzerland; 216 RWTH Aachen University, III. Physikalisches Institut A, Aachen, Germany; 217 University of Hamburg, Hamburg, Germany; 218 Isfahan University of Technology, Isfahan, Iran; 219 Brandenburg University of Technology, Cottbus, Germany; 220 Forschungszentrum Jülich, Juelich, Germany; 221 Physics Department, Faculty of Science, Assiut University, Assiut, Egypt; 222 Karoly Robert Campus, MATE Institute of Technology, Gyongyos, Hungary; 223 Institute of Physics, University of Debrecen, Debrecen, Hungary; 224 Institute of Nuclear Research ATOMKI, Debrecen, Hungary; 225 Universitatea Babes-Bolyai-Facultatea de Fizica, Cluj-Napoca, Romania; 226 MTA-ELTE Lendület CMS Particle and Nuclear Physics Group, Eötvös Loránd University, Budapest, Hungary; 227 Faculty of Informatics, University of Debrecen, Debrecen, Hungary; 228 Wigner Research Centre for Physics, Budapest, Hungary; 229 IIT Bhubaneswar, Bhubaneswar, India; 230 Institute of Physics, Bhubaneswar, India; 231 Punjab Agricultural University, Ludhiana, India; 232 UPES-University of Petroleum and Energy Studies, Dehradun, India; 233 Shoolini University, Solan, India; 234 University of Hyderabad, Hyderabad, India; 235 University of Visva-Bharati, Santiniketan, India; 236 Indian Institute of Science (IISc), Bangalore, India; 237 Indian Institute of Technology (IIT), Mumbai, India; 238 Deutsches Elektronen-Synchrotron, Hamburg, Germany; 239 Department of Physics, Isfahan University of Technology, Isfahan, Iran; 240 Sharif University of Technology, Tehran, Iran; 241 Department of Physics, University of Science and Technology of Mazandaran, Behshahr, Iran; 242 INFN Sezione di Bari, Università di Bari, Politecnico di Bari, Bari, Italy; 243 Italian National Agency for New Technologies, Energy and Sustainable Economic Development, Bologna, Italy; 244 Centro Siciliano di Fisica Nucleare e di Struttura Della Materia, Catania, Italy; 245 Scuola Superiore Meridionale, Università di Napoli Federico II, Naples, Italy; 246 Università di Napoli ‘Federico II’, Naples, Italy; 247 Consiglio Nazionale delle Ricerche-Istituto Officina dei Materiali, Perugia, Italy; 248 Riga Technical University, Riga, Latvia; 249 Consejo Nacional de Ciencia y Tecnología, Mexico City, Mexico; 250 IRFU, CEA, Université Paris-Saclay, Gif-sur-Yvette, France; 251 Institute for Nuclear Research, Moscow, Russia; 252 National Research Nuclear University ‘Moscow Engineering Physics Institute’ (MEPhI), Moscow, Russia; 253 Institute of Nuclear Physics of the Uzbekistan Academy of Sciences, Tashkent, Uzbekistan; 254 St. Petersburg Polytechnic University, St. Petersburg, Russia; 255 University of Florida, Gainesville, Florida, USA; 256 Imperial College, London, UK; 257 P.N. Lebedev Physical Institute, Moscow, Russia; 258 California Institute of Technology, Pasadena, CA USA; 259 Budker Institute of Nuclear Physics, Novosibirsk, Russia; 260 Faculty of Physics, University of Belgrade, Belgrade, Serbia; 261 Trincomalee Campus, Eastern University, Nilaveli, Sri Lanka; 262 INFN Sezione di Pavia, Università di Pavia, Pavia, Italy; 263 National and Kapodistrian University of Athens, Athens, Greece; 264 Ecole Polytechnique Fédérale Lausanne, Lausanne, Switzerland; 265 Universität Zürich, Zurich, Switzerland; 266 Stefan Meyer Institute for Subatomic Physics, Vienna, Austria; 267 Laboratoire d’Annecy-le-Vieux de Physique des Particules, IN2P3-CNRS, Annecy-le-Vieux, France; 268 Şırnak University, Sirnak, Turkey; 269 Near East University, Research Center of Experimental Health Science, Nicosia, Turkey; 270 Konya Technical University, Konya, Turkey; 271 Piri Reis University, Istanbul, Turkey; 272 Adiyaman University, Adiyaman, Turkey; 273 Necmettin Erbakan University, Konya, Turkey; 274 Bozok Universitetesi Rektörlügü, Yozgat, Turkey; 275 Marmara University, Istanbul, Turkey; 276 Milli Savunma University, Istanbul, Turkey; 277 Kafkas University, Kars, Turkey; 278 Istanbul Bilgi University, Istanbul, Turkey; 279 Hacettepe University, Ankara, Turkey; 280 Istanbul University-Cerrahpasa, Faculty of Engineering, Istanbul, Turkey; 281 Ozyegin University, Istanbul, Turkey; 282 Vrije Universiteit Brussel, Brussels, Belgium; 283 School of Physics and Astronomy, University of Southampton, Southampton, UK; 284 Rutherford Appleton Laboratory, Didcot, UK; 285 IPPP Durham University, Durham, UK; 286 Monash University, Faculty of Science, Clayton, Australia; 287 Università di Torino, Turin, Italy; 288 Bethel University, St. Paul, MN USA; 289 Karamanoğlu Mehmetbey University, Karaman, Turkey; 290 United States Naval Academy, Annapolis, MD USA; 291 Bingol University, Bingol, Turkey; 292 Georgian Technical University, Tbilisi, Georgia; 293 Sinop University, Sinop, Turkey; 294 Erciyes University, Kayseri, Turkey; 295 Institute of Modern Physics and Key Laboratory of Nuclear Physics and Ion-beam Application (MOE), Fudan University, Shanghai, China; 296 Texas A &M University at Qatar, Doha, Qatar; 297 Kyungpook National University, Daegu, Korea; 298 CERN, 1211 Geneva 23, Switzerland

## Abstract

Using a data sample of $\sqrt{s}=13\,\text {TeV}$ proton-proton collisions collected by the CMS experiment at the LHC in 2017 and 2018 with an integrated luminosity of $103\text {~fb}^{-1}$, the $\text {B}^{0}_{\mathrm{s}} \rightarrow \uppsi (\text {2S})\text {K}_\mathrm{S}^{0}$ and $\text {B}^{0} \rightarrow \uppsi (\text {2S})\text {K}_\mathrm{S}^{0} \uppi ^+\uppi ^-$ decays are observed with significances exceeding 5 standard deviations. The resulting branching fraction ratios, measured for the first time, correspond to ${\mathcal {B}}(\text {B}^{0}_{\mathrm{s}} \rightarrow \uppsi (\text {2S})K_\mathrm{S}^{0})/{\mathcal {B}}(\text {B}^{0}\rightarrow \uppsi (\text {2S})K_\mathrm{S}^{0}) = (3.33 \pm 0.69 (\text {stat})\, \pm 0.11\,(\text {syst}) \pm 0.34\,(f_{\mathrm{s}}/f_{\mathrm{d}})) \times 10^{-2}$ and ${\mathcal {B}}(\text {B}^{0} \rightarrow \uppsi (\text {2S})\text {K}_\mathrm{S}^{0} \uppi ^{+} \uppi ^{-})/ {\mathcal {B}}(\text {B}^{0} \rightarrow \uppsi (\text {2S})\text {K}^{0}_{\mathrm{S}}) = 0.480 \pm 0.013\,(\text {stat}) \pm 0.032\,(\text {syst})$, where the last uncertainty in the first ratio is related to the uncertainty in the ratio of production cross sections of $\hbox {B}^{0}_{\mathrm{s}}$ and $\hbox {B}^{0}$ mesons, $f_{\mathrm{s}}/f_{\mathrm{d}}$.

## Introduction

Decays of neutral  mesons into charmonium resonances ( , etc.) are well suited to study the flavour sector of the standard model (SM) and to search for indications of new physics beyond the SM. In the last decade, interest in  hadron decays to final states containing a charmonium resonance has increased after several exotic hadrons have been observed as intermediate resonances in multibody decays. Starting from the observation of *X*(3872) [1], many new charmonium-like states have been observed, such as *X*(4140) [2–5], *Y*(4260) [6, 7], and others, with properties (mass, width and decay pattern) not fitting into the landscape of traditional charmonium states. The first charged tetraquark candidate, $Z(4430)^{+}$ was discovered in the  decay as a peak in the  mass spectrum [8–11]. Many other exotic hadrons have been observed in the last 15 years [12, 13], and the nature of most of them is still unclear. Moreover, channels whose final state is accessible both from  and  can be used to measure time-dependent *CP* asymmetry [14–27] as well.

This paper presents the first measurement of the  and  decays, using a data sample of proton-proton collisions at $\sqrt{s}=13\,\text {TeV}$ collected by the CMS experiment at the CERN LHC in 2017 and 2018 with an integrated luminosity of 103 [28, 29]. Both decays can potentially be used for *CP* asymmetry measurements, and, in addition, the second one can also be used to search for intermediate exotic resonances. The  and  mesons are reconstructed using their decays into  and  , respectively. The  decay is chosen as the normalization channel for the measurement of the branching fractions, since its probability is precisely known [13], and its topology and kinematic properties are similar to those of the  or  decays. Therefore, using this normalization reduces the systematic uncertainties related to muon and track reconstruction. The relative branching fractions are measured using the relations1where  is the branching fraction, *N* is the number of reconstructed events in data, $\epsilon$ is the total reconstruction efficiency, and ${f_\mathrm {d}/f_\mathrm {s}}$ is the ratio of production cross sections of $\text {B}^{0}$ and $\text {B}^{0}_{\mathrm{s}}$ mesons (also called fragmentation fraction ratio). Charge-conjugate states are implied to be included throughout the paper.

Tabulated results are provided in the HEPData record for this analysis [30].

## The CMS detector and simulated event samples

The central feature of the CMS apparatus [31] is a superconducting solenoid of $6\,{\mathrm{m}}$ internal diameter, providing a magnetic field of $3.8\,{\mathrm{T}}$. Within the solenoid volume are a silicon pixel and strip tracker, a lead tungstate crystal electromagnetic calorimeter, and a brass and scintillator hadron calorimeter. Muons are measured in gas-ionization detectors embedded in the steel flux-return yoke outside the solenoid.

Events of interest are selected using a two-tiered trigger system [32]. The first level, composed of custom hardware processors, uses information from the calorimeters and muon detectors to select events at a rate of around $100\,{\mathrm{kHz}}$ within a time interval of less than $4\,\upmu \mathrm{s}$ [33]. The first-level trigger used in this analysis requires at least two muons. The second level, known as the high-level trigger, consists of a farm of processors running a version of the full event reconstruction software optimized for fast processing that reduces the event rate to around $1\,{\mathrm{kHz}}$ before data storage. The high-level trigger algorithm used in the analysis requires two opposite-sign muons compatible with the dimuon decay of a  meson with transverse momentum ( ) larger than $18\,\mathrm{GeV}$.

Simulated Monte Carlo samples for the decays of interest are generated for the analysis. The  8.230 package [34] with the CP5 tune [35] is used to simulate the production of the $\text {B}^{0}$ and $\text {B}^{0}_{\mathrm{s}}$ mesons, whose subsequent decays are performed by  1.6.0 [36], where final-state photon radiation is included using  3.61 [37, 38]. The lifetimes of $\text {B}^{0}$ and $\text {B}^{0}_{\mathrm{s}}$ mesons used in the generation are 1.52 and $1.47\,{\mathrm{ps}}$, respectively. The generated events are passed to a detailed GEANT4-based simulation [39] of the CMS detector, and are then processed using the same trigger and reconstruction as used for the collision data. The simulation includes effects from multiple proton-proton interactions in the same or nearby bunch crossings (pileup) with the multiplicity distribution tuned to match those of the data.

## Event reconstruction and selection

The reconstruction procedure starts with finding two muons of opposite charges, that must match those that triggered the event readout. The muon candidates are required to have , a pseudorapidity , and to satisfy general identification (soft-muon) criteria [40]. The two muons with a two-prong vertex fit probability  are paired to form the  candidate, which must have  and an invariant mass  (the world average  meson mass is  [13]).

The  candidates are formed from displaced two-prong vertices, as described in Ref. [41]. The  invariant mass is required to be within $\pm 20\,\text {MeV}$ of the world average value  [13], which corresponds to approximately three times the mass resolution. Selected  and  tracks are then refitted with their invariant mass constrained to  , and the obtained  candidate is required to have .

The  candidates are obtained through a kinematic vertex fit on the  system which constrains the dimuon mass to  . The  candidates are required to have , a 3D pointing angle between  and  to satisfy , and a transverse displacement significance for  of . Here  denotes the vector from the  production vertex to the  decay vertex, while  and  correspond to the length of , the transverse component of $\vec {D}$, and its uncertainty. To suppress the combinatorial background, additional requirements are applied: , , and , where the  meson transverse displacement  is calculated with respect to the primary vertex (PV). From all reconstructed proton-proton collision points, the PV is chosen as the one with the smallest  pointing angle, as in Refs. [42–44]. The pointing angle is the angle formed by the  candidate momentum and the vector from the PV to the reconstructed  candidate vertex. Furthermore, if in this procedure any of the tracks used in the  candidate reconstruction is included in the fit of the chosen PV, the track is removed, and the PV is refitted.

For the  candidates, two additional, oppositely charged, high-purity [45] tracks, assumed to be pions and having , are included in the  meson vertex fit, while the rest of the selection criteria are the same.

**Fig. 1:**
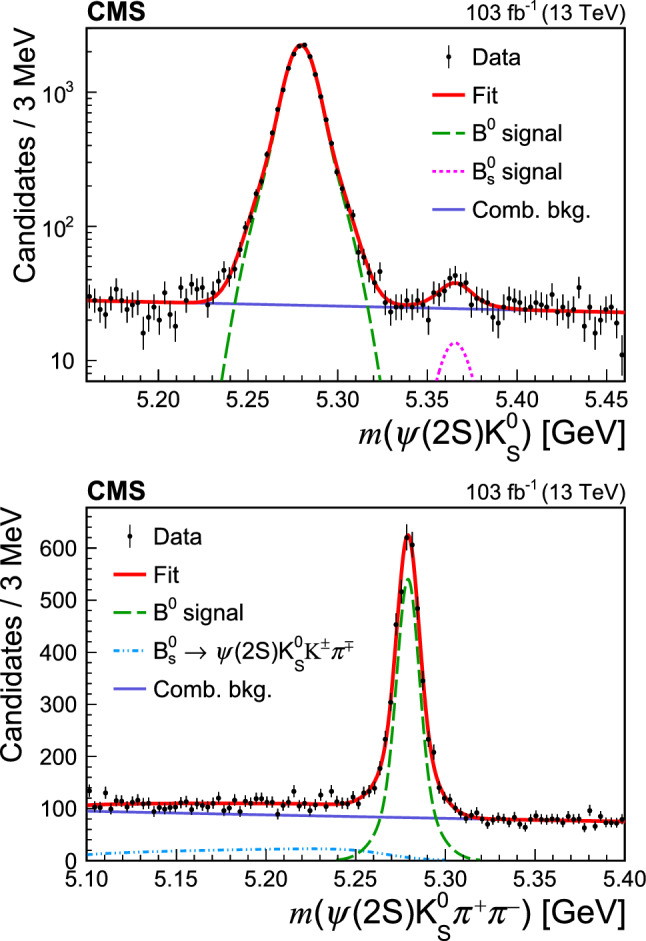
Measured invariant mass distributions of  (upper) and  (lower) candidates. The overlaid results from the fit are described in the text

## Observation of the $\text {B}^{0}_{\mathrm{s}} \rightarrow \uppsi (\text {2S})\text {K}^{0}_{\mathrm{S}}$ decay

The measured  invariant mass distribution is presented in Fig. 1 ( ). The $\text {B}^{0}$ signal (left peak) is described with a double Gaussian function with common mean, whose parameters are free to vary in an unbinned maximum-likelihood fit. It is found in simulation that the  signal (right peak) has the same shape as the  signal, but it is about 10% wider, because of the larger energy release in the decay. Therefore, the $\text {B}^{0}_{\mathrm{s}}$ signal is modelled with a double Gaussian function of the same shape as the $\text {B}^{0}$ signal, with the resolution parameters scaled by the ratio of the widths found in the simulation. The background is modelled with an exponential function. The good quality of the fit is verified by calculating the $\chi ^2$ between the binned distribution and the fit function, resulting in $\chi ^2=83$ for 91 degrees of freedom.

The ratio of signal yields  is extracted from the fit. Its uncertainty is calculated by taking into account the correlation between the uncertainties in $\text {B}^{0}_{\mathrm{s}}$ and $\text {B}^{0}$ yields, which are found to be $113 \pm 23$ and $16660 \pm 140$, respectively, where the uncertainties are statistical only.

The statistical significance of the  signal is evaluated with the likelihood ratio technique, comparing the background-only and signal-plus-background hypotheses, with the standard asymptotic formula [46], assuming that the conditions to apply Wilks’ theorem [47] are satisfied. For a significance estimation, the mass difference between the $\text {B}^{0}_{\mathrm{s}}$ and $\text {B}^{0}$ signals is fixed to the known value of $83.78\,\mathrm{MeV}$ [13]. The obtained significance is 5.2 standard deviations and varies in the range 5.1–5.4 standard deviations when accounting for the systematic uncertainties due to the choice of the fit model, discussed in Sect. 7.

## Observation of the $\text {B}^{0} \rightarrow \uppsi (\text {2S})\text {K}^{0}_{\mathrm{S}}\uppi ^{+}\uppi ^{-}$ decay

As shown in Fig. 1 ( ), the measured  mass distribution presents a clear  signal peak on top of a relatively small background. The $\text {B}^{0}$ signal is modelled with a double Gaussian function with common mean with all parameters free to vary, and the combinatorial background is described by an exponential function.

Studies of simulated events show that the  decay contributes to the reconstructed  mass distribution when the charged kaon is reconstructed as a pion. This relevant background contribution is accounted for in the fit to data by including a dedicated component with a freely varying normalization and a fixed shape that is obtained from simulation (Fig. 1,  ).

The signal yield  is found to be $3498 \pm 87$, where the uncertainty is statistical only. The $\chi ^2$ between the binned distribution and the fit function is 75 for 92 degrees of freedom, demonstrating the good quality of the fit. The significance of the  signal, evaluated as described in Sect. 4, exceeds 30 standard deviations.

**Fig. 2:**
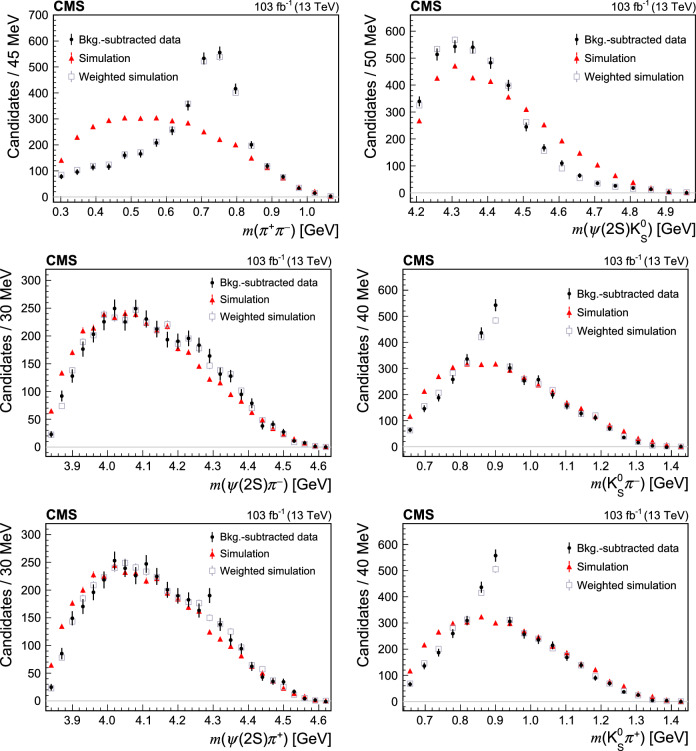
Distributions of 2-body intermediate invariant masses from the  decay. The data distributions (black dots) are background subtracted. Overlaid are the predictions of phase space simulations (red triangles), as well as the predictions after applying the reweighting procedure described in Sect. 7 (grey squares)

**Fig. 3:**
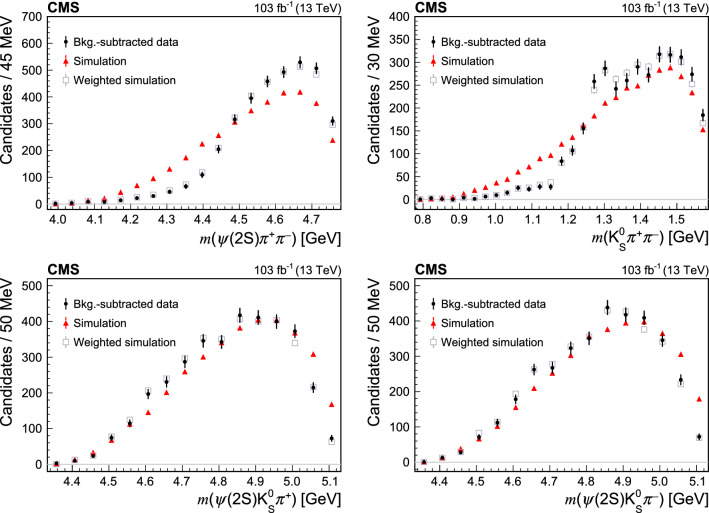
Distributions of 3-body intermediate invariant masses from the  decay. Data distributions (black dots) are background subtracted. Overlaid are the predictions of phase space simulations (red triangles), as well as the predictions after applying the reweighting procedure described in Sect. 7 (grey squares)

The intermediate invariant mass distributions, corresponding to the four-body  decay, are produced using the  [48] technique to subtract the non-$\text {B}^{0}$ background, using the  distribution fit described above. The correlations between the intermediate invariant masses and  have been checked to be below 10%. Figures 2 and 3 show the 2- and 3-body invariant mass distributions. Overlaid are the predictions of the 4-body phase space simulations, which provide poor description of the data since the simulations do not account for the intermediate resonance structure. The simulation after application of the reweighting procedure described in Sect. 7 is also shown. The mass distributions of  and one or two light mesons (, , , ) do not present any significant narrow peak that could indicate a contribution from an exotic charmonium state. The small excess at about 4.3 in the  distribution (Fig. 2, bottom left) is not significant, and there is no similar excess in the  distribution (Fig. 2, middle left). Moreover, exotic states previously found in this mass range are known to have large natural widths [12, 13]. Signs of the  (Fig. 2, middle and bottom right),  (Fig. 2, top left), and  (Fig. 3, top right) resonances are seen in the mass distributions of ,  , and , respectively.

## Efficiencies

The total reconstruction efficiency for each decay channel is evaluated using samples of simulated events. It is calculated as the number of reconstructed events divided by the number of generated events, and includes the detector acceptance, trigger, and candidate reconstruction efficiencies. Only the ratios of such efficiencies are needed to measure the ratios $R_{\mathrm{s}}$ and  , thus reducing the systematic uncertainties associated with muon and track reconstruction.

The obtained efficiency ratios are found to bewhere the uncertainties are statistical only and are related to the size of the simulated event samples. The first ratio is close to unity, as expected, while the second ratio is significantly greater than unity because of the presence of two additional tracks in the denominator. The lifetimes of heavy and light $\text {B}^{0}_{\mathrm{s}}$ meson eigenstates differ by about $0.2\,{\mathrm{ps}}$ [13], which can have an impact on the efficiency . It was verified that the corresponding variations of $\text {B}^{0}_{\mathrm{s}}$ lifetime result in negligible changes in the efficiency.

The validation of Monte Carlo samples is performed by comparing distributions of variables used in the event selection between simulation and background-subtracted data. No significant deviation is found, and thus no systematic uncertainties in the efficiency ratio are assigned related to data-simulation discrepancies in those variables.

## Systematic uncertainties

Many systematic uncertainties, related to the efficiency of the trigger as well as the reconstruction and identification of the muons, cancel out in the measured ratios $R_{\mathrm{s}}$ and  . Since the  and  decays have the same number of tracks in the final state, uncertainties related to the track reconstruction are of the same size and correlated, and therefore cancel out when propagated to the measured ratio $R_{\mathrm{s}}$. For the ratio  , we consider an additional uncertainty of 4.2% from the uncertainty in the tracking efficiency of two additional pions [49].

The systematic uncertainty related to the choice of the fit model is evaluated by testing different models. The largest deviation in the measured ratio from its baseline value is taken as a systematic uncertainty, separately for the variations of the signal and background models. Several alternative signal models were considered. One is a double Gaussian function for $\text {B}^{0}$ and $\text {B}^{0}_{\mathrm{s}}$ signals with the resolution shape fixed to the expectations taken from simulation with only the resolution scaling parameter being free in the fit. Another signal model is a Student’s *t*-distribution [50] with the value of the *n* parameter fixed to the one measured in simulation. Alternative background models include polynomials of the second and third degrees, an exponential multiplied by a polynomial, and a power function multiplied by an exponential, where in all cases the background shape parameters are free to vary in the fits.

**Table 1:** Systematic uncertainties (in %) of the measured branching fraction ratios

Source	$R_{\mathrm{s}}$	
Background model	2.5	0.8
Signal model	1.5	0.8
Shape of contribution	–	0.5
Finite size of simulation samples	1.3	1.1
Intermediate resonances	–	5.0
Tracking efficiency	–	4.2
Total	3.2	6.7

**Fig. 4:**
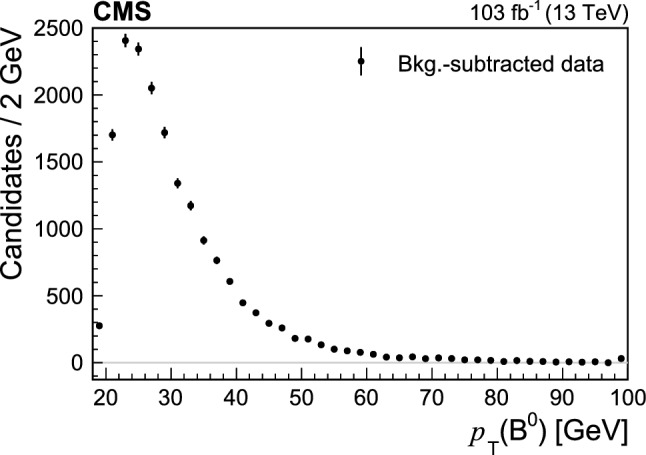
Background-subtracted  distribution in data for the  signal. The last bin includes the overflow

The uncertainty related to the finite size of the simulation samples (used to measure the efficiencies in Sect. 6) is also considered as a systematic uncertainty.

The uncertainty associated with the shape of the  contribution to the  invariant mass distribution is estimated by varying the shape parameters within their uncertainties. The largest deviation of  from the baseline value is 0.5% which is taken as a systematic uncertainty.

As discussed in Sect. 5, the simulation for the  decay does not take into account the intermediate resonance structure, leading to a significant disagreement between data and simulation in the 2- and 3-body mass distributions. This results in a potential bias in the efficiency reported in Sect. 6. To estimate the corresponding systematic uncertainty, the simulated sample is reweighted to be consistent with the data, and the difference between the baseline efficiency and the efficiency obtained on the weighted sample is taken as a systematic uncertainty. Due to the limited number of events, it is impossible to assign weights taking the ratio of data to simulation in bins of multi-dimensional phase space of the considered 4-body decay. An iterative procedure has been developed that operates with one-dimensional weights corresponding to each 2- and 3-body invariant mass, gradually making the mass distributions on weighted simulation sample closer and closer to data, until a satisfactory agreement in all intermediate invariant mass distributions is achieved. The distributions of invariant masses obtained with the weighted simulation sample are presented in Figs. 2 and 3. The efficiency obtained on the weighted simulation sample deviates from the baseline value by 5%, which is taken as a systematic uncertainty due to the intermediate resonance structure. This efficiency correction procedure with iterative reweighting is verified using a dedicated simulation sample instead of data, in which the contributions from  and  resonances are included with arbitrary magnitudes.

All uncertainties described above, excluding the one related to $f_{\mathrm{s}}/f_{\mathrm{d}}$ for the ratio $R_{\mathrm{s}}$, are summarized in Table 1 together with a total systematic uncertainty, calculated as a sum in quadrature of the individual sources.

A measurement of the ratio of the $\text {B}^{0}_{\mathrm{s}}$ and $\text {B}^{0}$ fragmentation fractions, $f_{\mathrm{s}}/f_{\mathrm{d}}$, in proton-proton collisions at the LHC has been recently reported by the LHCb Collaboration [51]: , where  in GeV is the transverse momentum of a  meson produced in $13\,\mathrm{TeV}$ proton-proton collisions. The ratio was found to be independent of the rapidity of the  meson, but with a significant dependence on the transverse momentum of the  candidate. The  distribution used in this analysis is shown in Fig. 4, where the background is subtracted using  . Using the LHCb result and the average  in our events of $31.2\,\mathrm{GeV}$, the $f_{\mathrm{s}}/f_{\mathrm{d}}$ value for the kinematic range of this analysis is obtained to be $f_{\mathrm{s}}/f_{\mathrm{d}} = 0.208 \pm 0.007$. The LHCb $f_{\mathrm{s}}/f_{\mathrm{d}}$ measurement is mostly dependent on the events with , while the majority of the events in this analysis have . Therefore, we assign an additional systematic uncertainty on $f_{\mathrm{s}}/f_{\mathrm{d}}$ as the difference between 0.208 and the value obtained under the assumption that $f_{\mathrm{s}}/f_{\mathrm{d}}$ becomes constant (0.2278) in the region . This additional uncertainty is estimated to be 0.020, and the total uncertainty on $f_{\mathrm{s}}/f_{\mathrm{d}}$ is obtained by summing it in quadrature with the uncertainty of 0.007 obtained above. The resulting fragmentation fraction ratio used in the $R_{\mathrm{s}}$ measurement is ${f_{\mathrm{s}}/f_{\mathrm{d}}=0.208\pm 0.021}$, with a relative uncertainty of 10%.

## Measured branching fractions

The branching fraction ratio of the  decay relative to the  one is measured using Eq. (1) to bewhere the last uncertainty is related to the used value ${f_{\mathrm{s}}/f_{\mathrm{d}}=0.208\pm 0.021}$. Since the knowledge of $f_{\mathrm{s}}/f_{\mathrm{d}}$ at large  can be updated with future measurements, allowing to improve the $R_{\mathrm{s}}$ evaluation, we also provide the measurement of the productIn addition, the transverse momentum distribution of the measured  candidates is presented in Fig. 4 and in the HEPData record for this analysis [30].

The branching fraction ratio of the  decay with respect to the  one is measured to beThis ratio is very close to the similar ratio measured with  instead of  [52].

Using the world average value  [13], the branching fractions of the two newly observed decays are evaluated:where the last uncertainties are from the uncertainty in .

## Summary

The  and  decays are observed using proton-proton collision data collected by the CMS experiment at $13\,\mathrm{TeV}$ with an integrated luminosity of 103 . Their branching fractions are measured with respect to the  decay to be , and , where the last uncertainty in the first ratio corresponds to the uncertainty in the ratio of production cross sections of $\text {B}^{0}_{\mathrm{s}}$ and $\text {B}^{0}$ mesons. The 2- and 3-body invariant mass distributions of the  decay products do not show significant exotic narrow structures in addition to the known light meson resonances. Further studies with more data will be needed to investigate more precisely the internal dynamics of the  decay, and to perform *CP* asymmetry measurements in the two observed decays in the future.

## Data Availability

This manuscript has no associated data or the data will not be deposited. [Authors’ comment: Release and preservation of data used by the CMS Collaboration as the basis for publications is guided by the CMS policy as stated in “CMS data preservation, re-use and open access policy” (https://cms-docdb.cern.ch/cgibin/PublicDocDB/RetrieveFile?docid=6032 &filename=CMSDataPolicyV1.2.pdf &version=2).”]
